# Supplementary material for: Identification of SARS-CoV-2 Main Protease Inhibitors Using Chemical Similarity Analysis Combined with Machine Learning
Source: Pharmaceuticals (Basel). 2024 Feb 12;17(2):240. doi: 10.3390/ph17020240 (PMC10892746; doi:10.3390/ph17020240)
Supplement: Supplementary file 1 [file pharmaceuticals-17-00240-s001.zip › pharmaceuticals-2846685-supplementary.pdf]

Table S1. Reference compounds for computational screening

Compound	Use	Activity on	Evidence	Reference
Acetophenazina	Antipsychotic	SARS-CoV	In vitro-cells	[50]
Aloxistatin	Research	HCoV-OC43, HCoV-NL63, MERS-CoV, MHV-A59	In vitro-cells	[52,57]
Amodiaquine	Antimalarial	MERS-CoV, SARS-CoV	In vitro-cells	[52,57]
Anisomycin	Research	MERS-CoV, SARS-CoV	In vitro-cells	[52,57]
Arbidol	Antiviral	MERS-CoV, SARS-CoV	In vitro-cells	[52]
Astemizole	Antihistamine	SARS-CoV-2	In vitro-cells	[52,57]
Baicalin	Research	MERS-CoV, SARS-CoV	In vitro-cells	[52,57]
Baricitinib	Antirheumatic	SARS-CoV	In vitro-cells	[50]
Chlorogenic acid	Research	MERS-CoV, SARS-CoV	In vitro-cells	[52]
Chloroquine	Antimalarial	MERS-CoV, SARS-CoV	In vitro-cells	[52]
Chlorphenoxamine	Retired	SARS-CoV	In vitro-cells	[52,57]
Chlorpromazine	Antipsychotic	SARS-CoV	In vitro-cells	[52]

Cinanserin	Research	MERS-CoV, SARS-CoV	In vitro-cells	[52,57]
Clomipramine	Antidepressant	MERS-CoV, SARS-CoV	In vitro-cells	[52,57]
Cycloheximide	Research	MERS-CoV	In vitro-cells	[52,53, 57]
Cytarabine	Antineoplastic	MERS-CoV, SARS-CoV	In vitro-cells	[52,53, 57]
Darunavir	Antiviral	MERS-CoV, SARS-CoV	In vitro-cells	[52,57]
Dasatinib	Antineoplastic	SARS-CoV-2	In vitro-cells	[52,57]
Dosulepin	Antidepressant	MERS-CoV	In vitro-cells	[54]
Emetin	Research	HCoV-OC43, HCoV-NL63, MERS-CoV, MHV-A59	In vitro-cells	[52,57]
Favipiravir	Antiviral	SARS-CoV-2	In vitro-cells	[56]
Fluphenazine	Antipsychotic	HCoV-OC43, HCoV-NL63, MERS-CoV, MHV-A59	In vitro-cells	[50]
Fluspirilene	Antipsychotic	MERS-CoV, SARS-CoV	In vitro-cells	[52,57]
Gemcitabine	Antineoplastic	MERS-CoV, SARS-CoV	In vitro-cells	[52]
Glycyrrhizin	Sweetener	MERS-CoV, SARS-CoV	In vitro-cells	[52,57]

Hydroxychloroquine	Antimalarial	SARS-CoV	In vitro-cells	[50]
Hydroxyzine	Antihistamine	MERS-CoV, SARS-CoV	In vitro-cells	[52]
Imatinib	Antineoplastic	MERS-CoV	In vitro-cells	[52]
Ivermectin	Antiparasitic	SARS-CoV-2	In vitro-cells	[58]
Loperamide	Antidiarrheal	MERS-CoV, SARS-CoV	In vitro-cells	[52,57]
Lycorine	Research	MERS-CoV	In vitro-cells	[52,57]
Mefloquine	Antimalarial	MERS-CoV	In vitro-cells	[52,57]
Methotrimeprazine	Antipsychotic	MERS-CoV, SARS-CoV	In vitro-cells	[52,57]
Mofetil	Immunosuppressant	MERS-CoV	In vitro-cells	[50]
Monensin	Antibacterial (vet)	HCoV-OC43, HCoV-NL63, MERS-CoV, MHV-A59	In vitro-cells	[50]
Mycophenolic acid	Immunosuppressant	HCoV-OC43, HCoV-NL63, MERS-CoV, MHV-A60	In vitro-cells	[50]
Niclosamide	Anthelmintic	SARS-CoV	In vitro-cells	[51]
Nitazoxanide	Antiprotozoal	SARS-CoV-2	In vitro-cells	[56]
Omacetaxine	Antineoplastic	MERS-CoV, SARS-CoV	In vitro-cells	[52]

Phenazopyridine	Anesthetic	HCoV-OC43, HCoV-NL63, MERS-CoV, MHV-A60	In vitro-cells	[50]
Promethazine	Antihistamine	MERS-CoV, SARS-CoV	In vitro-cells	[52,57]
Pyrvinium	Anthelmintic	MERS-CoV	In vitro-cells	[50]
Remdesivir	Antiviral	SARS-CoV	In vitro-cells	[56]
Sotrastaurin	Research	MERS-CoV, SARS-CoV	In vitro-cells	[52,57]
Tamoxifen	Antineoplastic	MERS-CoV, SARS-CoV	In vitro-cells	[52]
Terconazole	Antifungal	MERS-CoV, SARS-CoV	In vitro-cells	[52]
Thiethylperazine	Antiemetic	MERS-CoV	In vitro-cells	[50]
Thiothixene	Antipsychotic	MERS-CoV, SARS-CoV	In vitro-cells	[52]
Toremifene	Antineoplastic	MERS-CoV	In vitro-cells	[52,53, 57]
Trifluoperazine	Antidepressant	MERS-CoV, SARS-CoV	In vitro-cells	[52,57]
Triparanol	Retired	MERS-CoV, SARS-CoV	In vitro-cells	[52]
Valinomycin	Research	SARS-CoV	In vitro-cells	[51]
Lopinavir	Antiviral	HCoV-OC43, HCoV-NL63, MERS-CoV, MHV-A59	Clinical use	[50]

Ribavirin	Antiviral	MERS-CoV	Clinical use	[50]
Saracatinib	Research	MERS-CoV	Clinical use	[50]
6-Mercaptopurine	Antineoplastic	MERS-CoV	In vitro-free cells	[50]
6-Thioguanine	Antineoplastic	MERS-CoV	In vitro-free cells	[50]
Disulfiram	Prevent alcohol dependency	MERS-CoV	In vitro-free cells	[50]
N-ethylmaleimide	Research	MERS-CoV	In vitro-free cells	[50]
Nutlin-3A	Research	MERS-CoV	In vitro-free cells	[50]
Benztropine	Anticholinergic	SARS-CoV-2	Computational proposal	[55]

Table S2. Reference compounds with proven activity against Mpro and PLpro of SARS-CoV-2

Compound	IC_{50} against SARS-CoV-2 Mpro (µM)	IC_{50} against SARS-CoV-2 PLpro (µM)	Reference
Amodiaquine	No reported	19.85	[67]
Baicalin	6.41	177.6	[64, 69]
Baricitinib	25.31	No reported	[63]
Chlorogenic acid	29.48	0.54	[66]
Cinanserin	124.93	No reported	[62]
Glycyrrhizin	< 30	No reported	[61]
Ivermectin	21.53	No reported	[60]
Loperamide	No reported	33.5	[65]
Niclosamide	18.7	16.6	[59]
Tamoxifen	No reported	41.0	[59]
6-Mercaptopurine	No reported	21.6	[68]
6-Thioguanine	No reported	5.0	[68]
Disulfiram	2.1	No reported	[70]

Table S3. Compounds evaluated experimentally.

Internal ID	ZINC15 ID	SMILES	Supplier	Cat. number
1	ZINC66987	CN(C)C1=CC=C(C=C1)C2NC3=CC=CC4=C3C(=CC=C4)N2	Vitas M Chemical	STK005051
2	ZINC299345	C1=CC=C(C=C1)OC(=O)C2=CC=CC=C2OC(=O)C3=CC=CO3	Vitas M Chemical	STK388138
3	ZINC600324	COC1=CC2=C(C=C1)SCCN(C2)C(=O)CCN3CCC(CC3)CC4=CC=CC=C4	Sigma-Aldrich	SML0549
4	ZINC3313600	CN1CCCN(CC1)S(=O)(=O)C2=CC3=CC=CC=C3C=C2	Enamine	Z45547537
5	ZINC3861553	CN(C)C1=CC=C(C=C1)C(C2=CC=C(C=C2)N(C)C)C3=CC=C(C=C3)N(C)C	TargetMol	T1343
6	ZINC4272012	CN(C)C1=CC=C(C=C1)C(C2=CC=CC=C2)C3=CC=C(C=C3)N(C)C	MedChemExpress	HY-D0300
7	ZINC4759224	CN(C)C1=CC=C(C=C1)C(=O)N(CC2=CC=CC=C2)CC3=CC=CC=C3	Chembridge	P-7363096
10	ZINC8579480	CCN(CC1=CC2=CC=CC=C2NC1=O)C(=O)C3=CN=CC=C3	Vitas M Chemical	STL038212
11	ZINC9435742	CC1=CC2=C(C(=CC(=O)O2)C)C(=C1)OCC(=O)N3CCC(CC3)(C4=CC=C(C=C4)Cl)O	Vitas M Chemical	STK608237
13	ZINC13878776	CC1=CC(=C(C(=O)N1)[N+](=O)[O-])OC(=O)C2=CC3=CC=CC=C3C=C2	Life Chemicals Inc.	F1826-0081
13a	ZINC4248385	CC1=CC(=C(C(=O)N1)[N+](=O)[O-])OC(=O)C2=CC=CC3=CC=CC=C32	Life Chemicals Inc.	F1826-0080
13b	ZINC13523222	CC1=CC=C(C=C1)C(=O)OC2=C(C(=O)NC(=C2)C)[N+](=O)[O-]	Life Chemicals Inc.	F1826-0032
13c	ZINC4248365	CC1=CC(=C(C(=O)N1)[N+](=O)[O-])OC(=O)C2=CC=C(C=C2)Br	Life Chemicals Inc.	F1826-0059
14	ZINC19360158	COC1=CC=CC=C1CN2CCN(CC2)CC3=CC4=CC=CC=C4C=C3	Vitas M Chemical	STK145318
18	ZINC19782432	CC(C)CC(=O)N1CCN(CC1)CC2=CC3=CC=CC=C3C=C2	Asinex	P-596350389

Table S4. Molecular docking scores of compounds **13–13**c

Compound	Score (kcal/mol)
**13**	-6.83
**13a**	-6.81
**13b**	-6.41
**13c**	-6.67

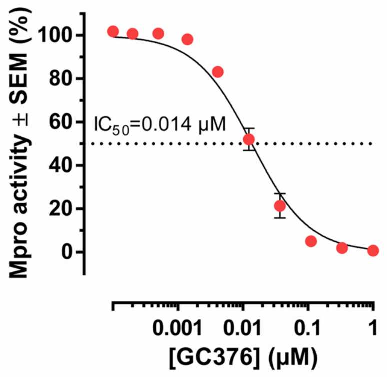

Figure S1. Concentration–response curve for the reported Mpro inhibitor GC376 in our FRET assay. A representative assay form two performed is shown.

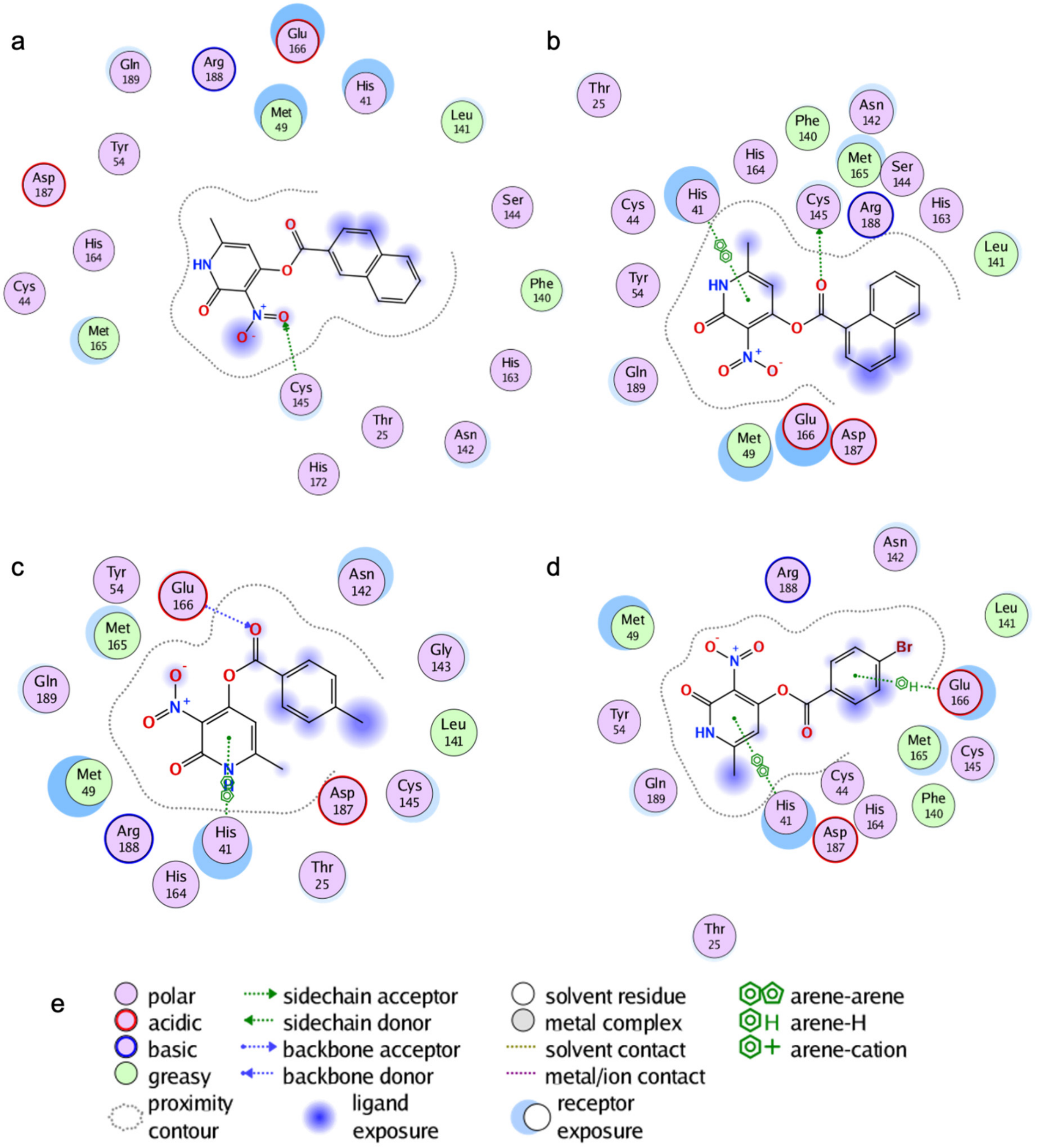

Figure S2. Binding mode of identified Mpro inhibitors predicted by molecular docking for the compound **13** (a), **13a** (b), **13b** (c), and **13c** (c). Nomenclature for two-dimensional interaction diagrams (e).

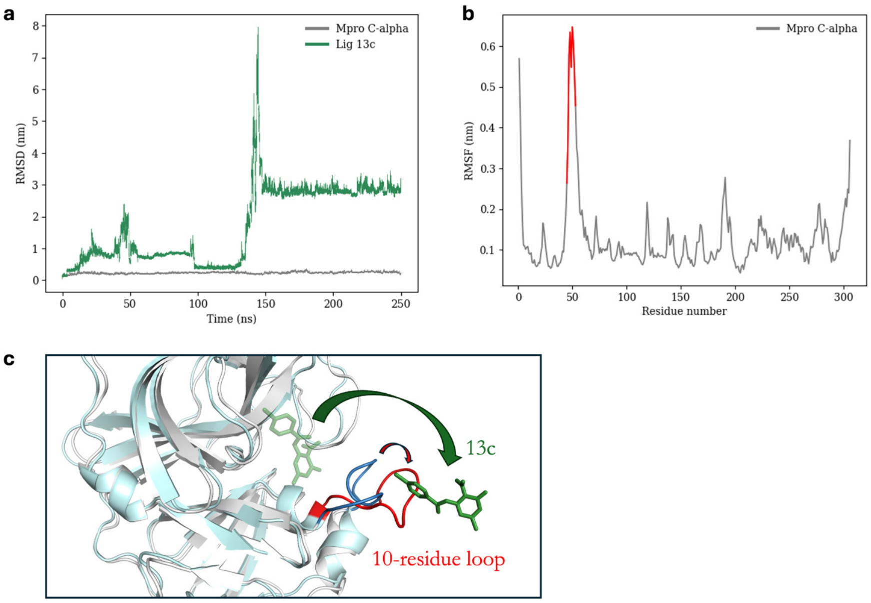

Figure S3. Molecular dynamics simulation of SARS-CoV-2 Mpro/13c complex. (a) Root Mean Square Deviation (RMSD) of ligand **13c** (green line) and Mpro (gray line). Mpro RMSD was 2.32 ± 0.31 Å. (b) Root Mean Square Fluctuation (RMSF) of Mpro alpha-carbons. Values for residues 43 to 54, corresponding to a loop with high fluctuation, are highlighted in red. c) Comparison of enzyme-ligand complex at initial conformation (Mpro: white cartoon, 43-54 loop: blue, ligand: transparent green) and after 45 ns of simulation (Mpro: pale cyan cartoon, 43-54 loop: red, ligand: solid green). Arrows indicate temporal evolution.

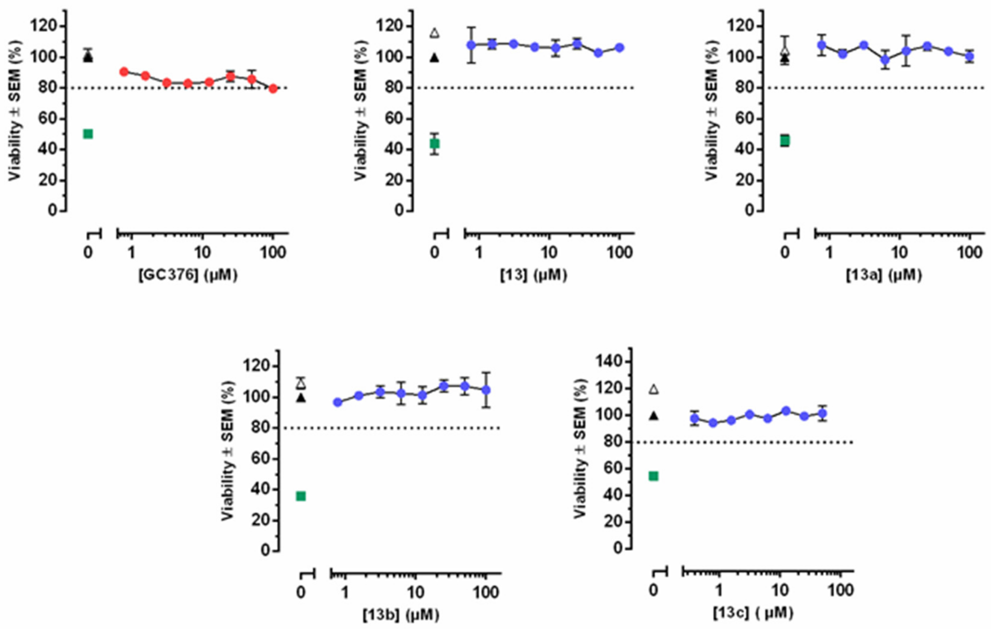

Figure S4. Cytotoxicity of SARS-CoV-2 Mpro inhibitors on CCL81 Vero cells exposed for 72 h to the selected compounds. Concentration-response curves for GC376 (0.078-10 µM), 13-13b (0.78-100 µM) and **13c** (0.39-50 µM). Data were normalized against the corresponding vehicle control (▲). Vehicle was DMSO 0.2% for GC376, **13**, **13a**, **13b**, and DMSO 0.5% for **13c**. Plots show the mean ± the standard error of the mean (SEM) from three independent experiments. All experiments included cells without treatment (△), and the cytotoxic drug doxorubicin 2.5 µM (■) as positive control.

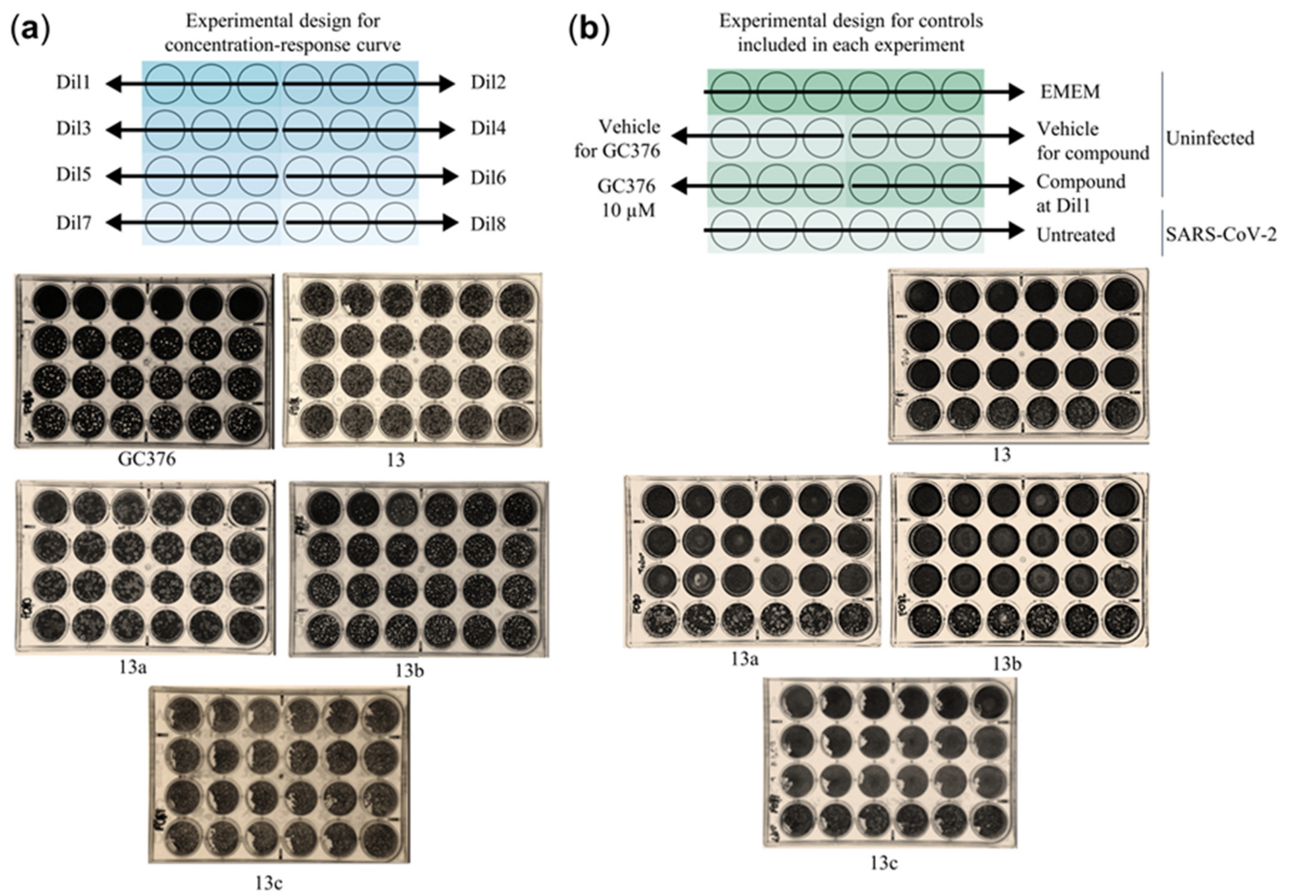

Figure S5. Cytopathic effect elicited by SARS-CoV-2 on cell cultures treated with Mpro inhibitors. (**a**) Representative Vero CCL8 cultures showing the cytopathic effect elicited by SARS-CoV-2 (72 h) in presence of compounds **13**–**13c** or the positive control GC376. The arrangement of the plate is shown at the top: Dil: dilution. (**b**) Representative Vero CCL8 cultures employed as controls. These control plates were run simultaneously than those shown in A.
